# Assessing visually guided reaching in people with multiple sclerosis with and without self-reported upper limb impairment

**DOI:** 10.1371/journal.pone.0262480

**Published:** 2022-01-21

**Authors:** Darrin O. Wijeyaratnam, Thomas Edwards, Lara A. Pilutti, Erin K. Cressman

**Affiliations:** 1 School of Human Kinetics, Faculty of Health Sciences, University of Ottawa, Ottawa, Ontario, Canada; 2 Interdisciplinary School of Health Science, Faculty of Health Sciences, University of Ottawa, Ottawa, Ontario, Canada; 3 Brain and Mind Research Institute, University of Ottawa, Ottawa, Ontario, Canada; Universita degli Studi di Napoli Federico II, ITALY

## Abstract

The ability to accurately complete goal-directed actions, such as reaching for a glass of water, requires coordination between sensory, cognitive and motor systems. When these systems are impaired, like in people with multiple sclerosis (PwMS), deficits in movement arise. To date, the characterization of upper limb performance in PwMS has typically been limited to results attained from self-reported questionnaires or clinical tools. Our aim was to characterize visually guided reaching performance in PwMS. Thirty-six participants (12 PwMS who reported upper limb impairment (MS-R), 12 PwMS who reported not experiencing upper limb impairment (MS-NR), and 12 age- and sex-matched control participants without MS (CTL)) reached to 8 targets in a virtual environment while seeing a visual representation of their hand in the form of a cursor on the screen. Reaches were completed with both the dominant and non-dominant hands. All participants were able to complete the visually guided reaching task, such that their hand landed on the target. However, PwMS showed noticeably more atypical reaching profiles when compared to control participants. In accordance with these observations, analyses of reaching performance revealed that the MS-R group was more variable with respect to the time it took to initiate and complete their movements compared to the CTL group. While performance of the MS-NR group did not differ significantly from either the CTL or MS-R groups, individuals in the MS-NR group were less consistent in their performance compared to the CTL group. Together these findings suggest that PwMS with and without self-reported upper limb impairment have deficits in the planning and/or control of their movements. We further argue that deficits observed during movement in PwMS who report upper limb impairment may arise due to participants compensating for impaired movement planning processes.

## Introduction

In order to interact with objects in our everyday environment, our central nervous system (CNS) must transform incoming sensory information (e.g., visual, haptic and proprioceptive input) into appropriate motor commands so that the action can be completed as desired (e.g., one is able to reach for a pen or a cup of coffee). Extensive work has been conducted to establish how visually guided reaching movements are planned and controlled in healthy individuals under various constraints [1–7]. These experiments have revealed that movement errors can be corrected in subsequent trials by engaging in offline planning, where offline planning processes occur before movement initiation and are presumed to be responsible for getting the hand in the vicinity of the target [4, 8–10]. As well, movement errors can be corrected during movement execution in order to reduce the error between one’s hand and target, thus demonstrating online control processes [11–15].

The contribution of offline planning and online control processes, and hence how movements are corrected, is determined by evaluating movement outcome, as well as kinematic variables related to reach performance. For example, measures such as the time it takes to initiate one’s movement (i.e., reaction time (RT)) and initial reach direction error (IDE) provide an indication of the engagement of offline planning processes [5, 6]. Movements with longer RT and lower IDE suggest that participants are taking longer to plan their movements to minimize early errors and to maximize endpoint accuracy. On the other hand, accuracy measures (i.e., resultant error) and the time required to execute a movement (i.e., movement time (MT)) provide an indication of the engagement of online control processes, such that small endpoint errors paired with movements long in duration (i.e., longer MT) suggest that participants may be making corrective movements during the reach to improve movement accuracy [9, 16]. Variability of the above measures provides further insight into an individual’s ability to incorporate sensory information during movement (i.e., online) and/or on subsequent trials (i.e., offline) to achieve task demands, such that increased variability is presumed to reflect a greater contribution of offline planning processes [6, 7, 17–19].

While we have gained much insight into the processes underlying goal-directed reaches in healthy individuals, less work has been done to establish patterns of upper limb performance in neurological pathologies, including people with multiple sclerosis (PwMS) [20, 21]. Multiple sclerosis (MS) is characterized by the presence of inflammatory demyelinated lesions distributed throughout the CNS [22, 23]. These lesions create structural and functional brain damage that affects sensory, cognitive and motor functions [24–26]. Today, imaging studies have improved our ability to detect structural and/or functional changes in the brain; however, our understanding of the impact of these neural changes on motor performance in PwMS remains limited, specifically with respect to visually guided reaching [27–29]. Previous investigations into upper limb function in PwMS have focused primarily on the detection and management of upper limb impairment using clinical tools, such as questionnaires (e.g., ABILHAND questionnaire) and tests of manual dexterity (e.g., 9-Hole Peg Test (9-HPT)) [30–35]. Additionally, previous work has prioritized the use of clinical disability scales (e.g., Expanded Disability Status Scale (EDSS)) that may not effectively capture upper limb impairment [30]. There is currently a need for more sensitive methods of evaluating the extent of upper limb impairment and functional loss in PwMS, as well as establishing where underlying deficits may arise (i.e., deficits in offline planning and/or online control processes). Furthermore, there is a need to determine how these deficits relate to subjective experiences of upper limb impairment, as consideration of self-reports of impairment have been implicated in upper limb assessment and been shown to benefit treatment for individuals with neurological pathologies (e.g., stroke; [36–38]).

Over the last two decades, robotic technology has become recognized as a promising tool to help characterize upper limb impairment, particularly in individuals with neurological pathologies, as it allows one to objectively evaluate motor behavior and identify underlying deficits [5, 39]. To date, robotic technology has been used extensively with individuals post-stroke, enabling researchers to assess and develop targeted treatment for deficits linked to offline planning and online control [39–49]. More recently, robotic technology has been incorporated into upper limb assessments and therapies in PwMS [50–58]. This volume of work in PwMS has used a variety of reaching tasks, which range from simple point-to-point movements [50] to reaching in different mechanical environments [52, 59]. Overall, results indicate that PwMS move more slowly [60], have more curved and less smooth movements [52, 53, 59, 61, 62], and reach with greater initial errors [51, 59, 61] compared to control participants without MS. These results have been observed in PwMS who fall at either end of the disability spectrum, experiencing either mild to no disability (i.e., EDSS < 1.0) or severe disability (i.e., EDSS > 6) based on EDSS scores.

The current experiment compared movement planning and control in PwMS during a visually guided reaching task to control participants without MS. We further evaluated whether offline planning and online control processes engaged during visually guided reaching differ between PwMS who self-report (i.e., MS-R) and who do not report (i.e., MS-NR) experiencing upper limb impairment, ensuring a range in participants’ subjective impairment experience. We hypothesized that PwMS who report upper limb impairment (MS-R) would show distinctive deficits in the processes underlying reaching (i.e., planning and control) compared to both people without MS (CTL) and PwMS who do not report upper limb impairment (MS-NR). Specifically, deficits in movement planning would be reflected by longer and less-consistent movement initiation times, as well as larger errors early in the trajectory in the MS-R group compared to both the MS-NR and CTL groups. Deficits in movement control would be reflected by longer and less-consistent movement durations in the MS-R group compared to both the MS-NR and CTL groups. We further hypothesized to observe deficits in movement planning and control in the MS-NR group compared to the CTL group, as previous studies investigating movements of the lower limb have reported reduced stability, decreased movement speed, and increased movement variability even in PwMS with mild disability levels [63–68].

## Methods

### Participants

This project is part of a larger study characterizing upper limb performance in PwMS across a variety of tasks differing in engagement of sensory, cognitive, and motor processes. In the current experiment, 36 participants completed a visually guided reaching task. The MS sample was recruited from MS-specific community outlets (e.g., the MS Society of Canada outlets and MS support groups). MS diagnosis was determined by self-report during the screening process. PwMS were asked to report whether they experienced difficulty performing daily tasks with either their dominant or non-dominant upper limbs. Those who stated that they had difficulty using their upper limbs were placed in Group 1 (i.e., MS-R; *n* = 12), whereas those who did not report difficulty using their upper limbs were placed in Group 2 (i.e., MS-NR; *n* = 12). The third group of participants consisted of age- and sex-matched controls (CTL; *n* = 12). In order to be eligible for inclusion, participants must have been: (1) between the ages of 18 and 65; and (2) willing to visit the research lab for two testing sessions. Additionally, PwMS must have: (1) a self-reported diagnosis of MS; (2) been relapse free for 30 days; and (3) experienced no changes to disease-modifying therapies in the past 6 months to be eligible to participate. This experiment was approved by the University of Ottawa Health Sciences and Science Research Ethics Board. All participants provided written informed consent and were informed that they could withdraw from the experiment at any time.

### Demographic and clinical characteristics

Demographic and clinical characteristics were collected during an initial testing session using a battery of self-reported questionnaires and/or clinical scales and tests. Height and weight were measured in the laboratory to the nearest 0.1 cm and 0.1 kg, respectively, using a scale with a stadiometer (Sartorius AG, Göttingen, Germany). Disability status was determined by the Expanded Disability Status Scale (EDSS), administered by a Neurostatus-certified assessor (TE, Level C). Upper limb performance was assessed using the 9-HPT (Patterson Medical, Warrenville, IL). The 9HPT involves participants picking up nine pegs, placing them in a peg board one at a time, and then removing the pegs from the holes one at a time [31]. Participants completed the 9-HPT on two consecutive trials with their dominant hand followed by two consecutive trials with their non-dominant hand. On all trials, participants were asked to complete the task as quickly as possible. The total time required to complete each trial in seconds was recorded and then averaged across trials completed with the same hand.

### Experimental apparatus: Visually guided reaching task

**Fig 1:**
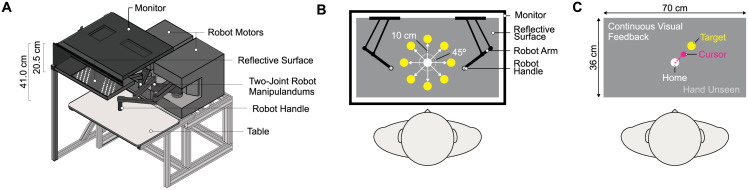
Experimental apparatus, dimensions and reaching environment. **(A)** Diagonal view of a cross-section of the experimental apparatus. Half of the monitor and reflective surface are displayed in order to show the right robot handle. **(B)** Visually guided reaching task. With full vision, participants reached from a central start position to one of eight peripheral targets (yellow circles) located 10 cm away from the start position (white circle) and distributed around the circumference of a circle (targets were separated by 45°) **(C)** Example of continuous visual feedback of the cursor throughout each reaching trial.

Testing took place in a secluded testing room with a two-joint robot manipulandum (Kinarm End-Point Lab, Kinarm, Kingston, ON, Canada). The Kinarm End-Point Lab consists of a downward facing monitor (EzSign model 47LD452B, LG. Seoul, South Korea; refresh rate = 60 Hz, workspace = 70 cm x 36 cm), which is located 20.5 cm above a reflective surface and 41.0 cm above two robotic handles (see Fig 1A). Thus, visual stimuli presented on the monitor are reflected by the surface and appear to lie in the same horizontal plane as the robot handles. Prior to each testing session, calibration of the Kinarm was carried out according to the manufacturer’s instructions.

During testing, participants were seated in a height-adjustable chair. Once participants were comfortably seated, the chair was locked in place and the lights were turned off. Participants then grasped a single handle of the two-joint robot with their dominant or non-dominant hand. Participants’ view of their limbs was obstructed by the reflective surface, as well as a black cloth placed between their shoulders and the experimental apparatus. The location of the robotic handle (and hence participants’ hands) was tracked at 1000 Hz, with a spatial accuracy of 0.1 mm.

### Procedure

Participants completed a visually guided reaching task with both their dominant and non-dominant hands in one experimental session (see Fig 1B). In general, participants were asked to comfortably reach with the robot handle from a central start position (white circle, 2 cm in diameter) to one of eight peripheral targets (yellow circle, 2 cm in diameter). The targets were 10 cm away from the start position and separated by 45° around the circumference of a circle. A trial started with the robot passively moving the participant’s hand to the start position. Following a 500 ms delay, one of the eight peripheral targets appeared and served as the “go signal” for participants to perform their action. Participants then reached out with either their dominant or non-dominant hand. The position of the handle (and hence their hand) was represented by a cursor on the reflective surface (magenta circle, 1 cm in diameter). A trial ended once the middle of the cursor (i.e., participant’s hand) was within 0.5 cm of the center of the target. Therefore, in order for a trial to be completed, participants were required to land the cursor on the target. This ensured that all participants received visual and proprioceptive (i.e., felt hand position) feedback associated with a successful reach.

Once the target was attained, the target and cursor disappeared, and the robot passively moved the participant’s hand back to the start position along a linear path within a movement time of 1000 ms. If participants attempted to move out from the linear path, a proportional resistance force was generated to maintain the linearity of the movement. Once the hand was positioned in the home position, the home position and cursor became visible again, signaling the start of the next trial (Fig 1C). Participants began with three practice trials. These trials were not included in our analyses. Following the practice trials, participants completed 8 blocks of 8 trials per hand for a total of 128 trials (i.e., 64 trials per hand). Within each block of trials, each target was presented once. Total task duration was approximately 15 minutes. The order with respect to which hand completed the task first (i.e., dominant versus non-dominant) was counterbalanced across participants.

### Data analysis

Reaching performance on all trials was analyzed post-experiment using custom written MATLAB scripts (Matlab R2013b (8.2.0.701), The MathWorks, Inc.). As indicated above, during the experiment, participants were required to get the cursor to the target. However, during post-experiment processing of reaching performance, the start and end of each movement were selected based on a velocity criterion, similarly to previous work [46]. In the current experiment, we use a velocity threshold whereby movement start was the time at which velocity first increased above 0.01 m/s and remained above 0.01 m/s for the subsequent 100 ms, while movement end was the time at which velocity first fell below 0.01 m/s. This post-experiment processing ensured that only participants’ initial reach was included in the analysis, rather than multiple corrective movements (i.e., participants stopping and then re-starting their movement). Endpoint position data were then used to screen for outliers. In particular, if a participant’s endpoint position in the horizontal or vertical direction was greater than 3 standard deviations above their respective mean endpoint position in the same dimension, the trial was removed from further analyses [69]. This screening resulted in the removal of 44 trials (0.95% of all trials).

We looked to quantitatively compare reaching performance with respect to offline planning and online control across PwMS (MS-NR and MS-R) and control participants when reaching with the dominant and non-dominant hands. We started by evaluating overall accuracy at movement endpoint by examining resultant error (RE) and absolute angular error (|EPAE|). RE was defined as the absolute distance of the cursor from the target at movement end. |EPAE| was defined as the difference between a movement vector joining the midpoint of the home position to the midpoint of the cursor position at the end of the movement and a reference vector joining the midpoint of the home position to the midpoint of the target location.

We further looked to determine the engagement of offline planning processes using reaction time (RT), peak velocity (PV), proportional time to peak velocity (pTTPV) and absolute initial reach direction errors (|IDE|). RT was defined as the time required to initiate a response from target presentation until the start of the movement. PV was defined as the greatest resultant velocity achieved during the movement, while pTTPV was expressed as the time PV was achieved as a percentage of overall MT (the time from movement initiation until movement end). |IDE| was defined as the difference between a movement vector (from the home position to the position of the cursor at peak velocity) and a reference vector joining the home position to the target location. We also looked to examine the engagement of online control processes using movement time (MT), path length (PL) and absolute change in angular error (|ΔAE|). MT was defined as the time required to execute the movement. PL was defined as the length of the reaching trajectory. |ΔAE| was defined as the difference between errors later in the movement compared to early in the movement (i.e., |ΔAE| = |EPAE—IDE|). Mean performance and variability (i.e., SD) were determined for each of the above measures. Absolute values are reported for EPAE, IDE and ΔAE, in order to examine changes in the magnitude of errors experienced early vs. late in reaching. All measures described above have previously been used to characterize upper limb performance in populations with neurological disorders (e.g., stroke research; [46]) and in healthy controls (for reviews see [5, 6]; c.f. [70]).

To determine if any of the within-trial measures differed across groups when reaching with either the dominant or non-dominant hands, the above variables were analyzed using a 3 Group (i.e., CTL, MS-NR, MS-R) *x* 2 Hand (i.e., non-dominant hand versus dominant hand) mixed analysis of variance (ANOVA) with repeated measures on the last factor in SPSS (IBM SPSS Statistics for Windows, Version 26, Armonk, NY). If the Mauchly’s test of sphericity was significant (*p* < 0.05), then the Greenhouse-Geisser correction factor was applied, and the adjusted *p*-values reported. Differences with a probability of 0.05 were considered statistically significant. Following a significant interaction, a simple effect analysis was conducted using a Bonferroni correction. The mean and variability of all dependent measures across Groups and with respect to both Hands are reported in the S1 File.

## Results

### Participants

A summary of the sample demographics and clinical characteristics are presented in Table 1. For MS participants, mean disease duration was 12.3 years ± 7.9 years for MS-NR participants and 16.4 years ± 9.2 years for MS-R participants. The distribution of MS types differed between the MS-NR and MS-R groups, such that majority of participants in the MS-NR group had relapsing remitting MS (83.4%), while more participants in the MS-R group had progressive MS (58.3%). There were no significant differences in age, height, or weight across the groups (all *p* > 0.05), and all groups had the same distribution of females (66.7%) and males (33.3%). The median EDSS score differed significantly between the MS-NR (*x̃* = 3.3, IQR = 2.0) and MS-R groups (*x̃* = 5.8, IQR = 2.5, *p* = 0.009), such that MS-NR participant’ scores corresponded to moderate disability while MS-R participant scores corresponded to severe disability. Moreover, the MS-R group took longer to complete the 9-HPT with their dominant hand compared to both the MS-NR and CTL groups (both *p* < 0.012). However, when completing the 9-HPT with their non-dominant hand, MS-R participants took significantly more time compared to CTL participants (*p* = 0.008), but not compared to MS-NR participants (*p* = 0.289).

**Table 1:** Demographic and clinical characteristics for the three experimental groups. Values reported as mean (standard deviation), unless specified otherwise.

	CTL	MS-NR	MS-R
**Demographic Characteristics**			
Age, years	49.5 (9.8)	46.3 (9.9)	49.3 (10.3)
Height, cm	170.4 (9.4)	169.1 (8.3)	165.4 (13.1)
Weight, kg	79.4 (12.6)	75.7 (12.8)	67.7 (10.3)
% Female	66.7%	66.7%	66.7%
**Clinical Characteristics**			
Disease Duration, years	-	12.3 (7.9)	16.4 (9.2)
MS Type			
Relapsing remitting, %	-	83.4%	41.7%
Primary progressive, %	-	8.3%	41.7%
Secondary progressive, %	-	8.3%	16.6%
EDSS ^†^ ^a^	-	3.3 (2.0)	5.8 (2.5)
9-HPT (dominant), seconds ^b^	17.5 (1.4)	22.5 (3.2)	29.4 (8.4)
9-HPT (non-dominant), seconds ^c^	19.0 (1.5)	24.9 (4.8)	31.3 (15.2)

Note. EDSS, Expanded Disability Status Scale; 9-HPT, 9-hole peg test.

^†^ Denotes values reported as median (interquartile range).

^a^ Denotes significant difference between MS-NR and MS-R.

^b^ Denotes significant difference between MS-R and the two other groups: MS-NR and CTL.

^c^ Denotes significant difference between MS-R and CTL.

### Sample reaching characteristics

**Fig 2:**
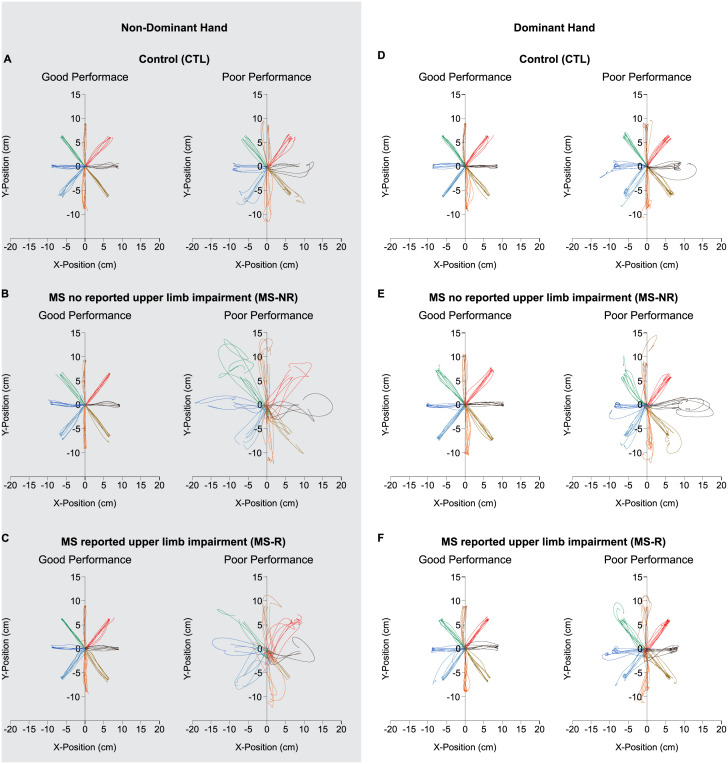
Sample reaching trajectories. Reaches completed to the 8 visual targets with the non-dominant (shaded area) and dominant hands. Each colour represents a reach trajectory to one of the 8 targets. Performance were classified as “Good” versus “Poor” based on their average path length (i.e., Good performance was represented as shorter path lengths versus Poor performance was represented as longer path lengths).

Reaching movements from exemplar participants are displayed in Fig 2. While performance differed across participants, all participants were able to get the cursor onto the target as required (i.e., complete the task). This indicates that PwMS, whether they reported upper limb impairment or not, were able to complete a simple reaching task to a visual target. To illustrate how reaches varied across participants, we present data for “Good” versus “Poor” participant performance for each of the 3 groups (see Fig 2). We defined Good and Poor performance based on PL, such that Good performance was represented by reaches by a participant within the group with the shortest average PL, and Poor performance was represented by reaches by a participant within the group with the longest average PL. While there was no difference in average PL across groups (MS-R = 9.7 cm ± 0.2 cm; MS-NR = 10.0 cm ± 0.2 cm; CTL = 9.4 cm ± 0.2 cm; Group: *F*(2,33) = 1.572, *p* = 0.223, η^2^ = 0.087), or PL variability (*x̄* = 1.3 cm ± 0.2 cm; Group: *F*(2,33) = 1.572, *p* = 0.223, η^2^ = 0.087), from Fig 2 we do see differences in the reaching trajectories across and within groups of participants, as reported below.

### General movement outcomes

Given that participants were required to land on the target, it is not surprising that accuracy was similar across groups. Analysis of RE indicated that endpoint errors were on average 1.4 cm across all participants when reaching with the dominant and non-dominant hands (Group: *F*(2,33) = 1.000, *p* = 0.379, η^2^ = 0.057; Hand: *F*(1,33) = 0.739, *p* = 0.396, η^2^ = 0.022). Similarly, variability of RE was comparable across groups, regardless of which hand participants reached with (Group: *F*(2,33) = 0.750, *p* = 0.480, η^2^ = 0.043; Hand: *F*(1,33) = 0.604, *p* = 0.443, η^2^ = 0.018). Analysis of |EPAE| revealed a significant main effect of Hand (*F*(1,33) = 9.127, *p* = 0.005, η^2^ = 0.217), such that angular errors were slightly greater at the end of the movement when participants reached with their non-dominant hand (*x̄* = 3.1° ± 0.3°) compared to their dominant hand (*x̄* = 2.5° ± 0.1°); however, there was no significant difference between groups (*F*(2,33) = 2.321, *p* = 0.114, η^2^ = 0.123). Variability of |EPAE| also differed between reaches with the dominant hand versus non-dominant hand (*F*(1,33) = 6.869, *p* = 0.013, η^2^ = 0.172), such that |EPAE| variability was higher when participants reached with their non-dominant hand (*x̄* = 2.8° ± 0.3°) compared to their dominant hand (*x̄* = 2.1° ± 0.2°). Again, performance did not differ across groups (*F*(2,33) = 1.668, *p* = 0.204, η^2^ = 0.1092).

### Offline movement planning

**Fig 3:**
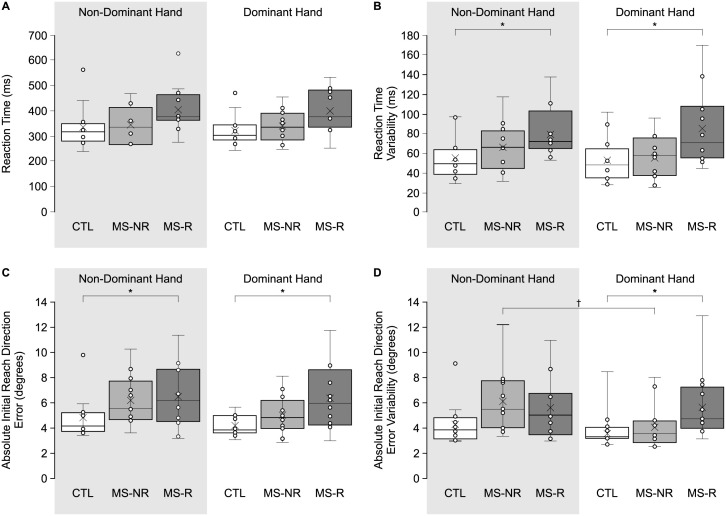
Box-and-whisker plots of offline movement planning measures. **(A-B)** Mean and variability of reaction time (ms). **(C-D)** Mean and variability of absolute initial reach direction error (degrees). *Shaded area* represents reaches performed with the non-dominant hand. *White bars* represent reaching performance by control participants (CTL), *light grey bars* represent reaching performance by PwMS who reported no upper limb impairment (MS-NR) and *dark grey bars* represent reaching performance by PwMS who reported upper limb impairment (MS-R). *Error bars* represent standard error of mean. *Asterisks* (*) represent significant differences between MS-R and CTL (*p* < 0.05). *Dagger* (†) represent a significant difference between Hands (*p* < 0.05).

In Fig 3, mean and variability of performance measures related to offline movement planning are presented. Fig 3A and 3B display mean and variability of RT. Analysis of mean RT revealed no significant main effects (both *p* > 0.068) or interactions (*p* = 0.871), which suggests that participants were spending a similar amount of time planning their movements. Analysis of RT variability revealed a significant main effect of Group (*F*(2,33) = 4.243, *p* = 0.023, η^2^ = 0.205), such that participants in the MS-R group were more variable in the time they took to initiate their movements compared to participants in the CTL group, regardless of hand used. RT variability in the MS-NR group did not differ significantly from either the MS-R (*p* = 0.132) or CTL groups (*p* = 1.000).

Analysis of PV indicated that PV achieved was comparable across groups, regardless of hand used (*x̄* = 0.23 m/s ± 0.01 m/s; Group *F*(2,33) = 0.556, *p* = 0.579, η^2^ = 0.033; Hand F value). Analysis of PV variability also indicated that performance was comparable across Groups (*x̄* = 0.046 m/s ± 0.004 m/s; Group *F*(2,33) = 0.314, *p* = 0.733, η^2^ = 0.019). Furthermore, participants spent a similar proportion of their movement achieving PV (i.e., pTTPV) when reaching with either the dominant or non-dominant hands (*x̄* = 53.0% ± 1.3%; Group: *F*(2,33) = 0.268, *p* = 0.767, η^2^ = 0.016; Hand: *F*(1,33) = 1.625, *p* = 0.211, η^2^ = 0.047). Participants were also comparable in the consistency regarding the time PV that was achieved (i.e., SD pTTPV) when reaching with either the dominant or non-dominant hands (*x̄* = 14.5% ± 0.5%; Group: *F*(2,33) = 0.336, *p* = 0.717, η^2^ = 0.020; Hand: *F*(1,33) = 2.773, *p* = 0.105, η^2^ = 0.078).

Mean and variability of |IDE| are shown in Fig 3C and 3D. Analysis of |IDE| revealed a significant main effect for Group (*F*(2,33) = 3.821, *p* = 0.032, η^2^ = 0.188), such that the MS-R group had larger |IDE| (*x̄* = 6.4° ± 0.5°) compared to the CTL group (*x̄* = 4.5° ± 0.5°). Again, initial errors observed for the MS-NR group (*x̄* = 5.6° ± 0.5°) did not differ from those observed in the MS-R and CTL groups (*p* > 0.377). Analysis of the variability of the |IDE| revealed a significant interaction between Group *x* Hand (*F*(2,33) = 0.448, *p* = 0.019, η^2^ = 0.212), with post hoc analysis revealing that the MS-R group demonstrated greater variability in |IDE| when reaching with the dominant hand compared to the CTL group (*p* = 0.013). Variability of |IDE| observed in the MS-NR group did not differ from either the MS-R or CTL groups (*p* > 0.081), but MS-NR participants did demonstrate greater variability in |IDE| when reaching with their non-dominant hand (*x̄* = 6.1° ± 0.7°) compared to their dominant hand (*x̄* = 4.1° ± 0.5°; *p* < 0.001).

### Online movement control

**Fig 4:**
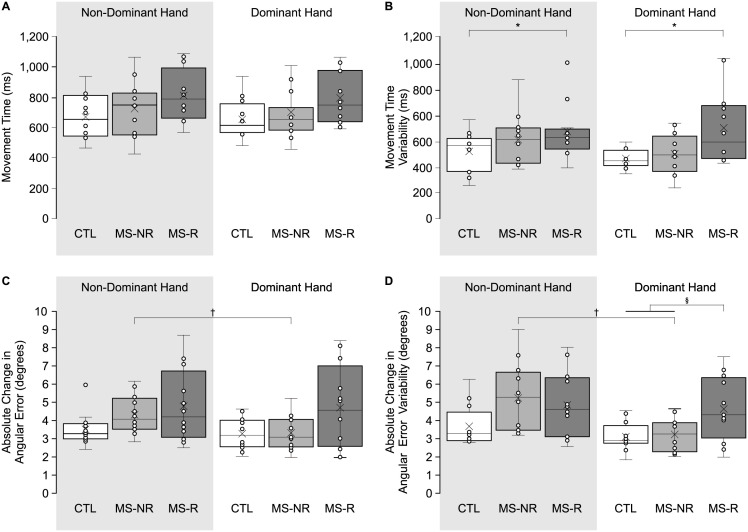
Box-and-whisker plots of online movement control measures. **(A-B)** Mean and variability of movement time (ms). **(C-D)** Mean and variability of absolute change in angular errors (degrees). *Shaded area* represents reaches performed with the non-dominant hand. *White bars* represent reaching performance by control participants (CTL), *light grey bars* represent reaching performance by PwMS who reported no upper limb impairment (MS-NR) and *dark grey bars* represent reaching performance by PwMS who reported upper limb impairment (MS-R). *Error bars* represent standard error of mean. *Asterisks* (*) represent significant differences between MS-R and CTL (*p* < 0.05). *Dagger* (†) represent a significant difference between Hands (*p* < 0.05). Section (§) represent a significant difference between the MS-R group and the two other groups (i.e., MS-NR and CTL).

In Fig 4, mean and variability of performance measures related to online movement control are presented. Fig 4A and 4B display mean and variability of MT. Average MT was 726.7 ms ± 26.4 ms, which did not differ across Groups (*F*(2,33) = 2.295, *p* = 0.107, η^2^ = 0.127), or between Hands (*F*(1,33) = 2.037, *p* = 0.163, η^2^ = 0.058), indicating that all participants reached with similar MT. Analysis of MT variability revealed a main effect of Group (*F*(2,33) = 4.211, *p* = 0.024, η^2^ = 0.203), such that participants in the MS-R group demonstrated more variable MT compared to participants in the CTL group. MT for the MS-NR group did not differ from the MS-R and CTL groups (*p* > 0.207).

Mean and variability of |ΔAE| from PV to EP are shown in Fig 4C and 4D. Participants slightly changed their reaching trajectories (*x̄* = 4.0° ± 0.2°) throughout the movement. ANOVA revealed a main effect of Group (*F*(2,33) = 3.173, *p* = 0.055, η^2^ = 0.161), and post hoc analysis approached statistical significance such that slightly greater changes were observed in the MS-R group (*x̄* = 4.8° ± 0.4°) compared to the CTL group (*x̄* = 3.4° ± 0.4°, *p* = 0.058). We also observed a main effect of Hand (*F*(1,33) = 4.626, *p* = 0.039, η^2^ = 0.123), such that participants’ |ΔAE| when reaching with their non-dominant hand (*x̄* = 4.2° ± 0.2°) was greater than when reaching with their dominant hand (*x̄* = 3.8° ± 0.3°). Analysis of |ΔAE| variability revealed a Group *x* Hand interaction (*F*(2,33) = 7.007, *p* = 0.003, η^2^ = 0.298), and post hoc analysis indicated that the variability of changes in reaches for the MS-R group (*x̄* = 4.6° ± 0.4°) was greater than the MS-NR (*x̄* = 3.2° ± 0.4°; *p* = 0.028) and CTL (*x̄* = 3.1° ± 0.4°; *p* = 0.016) groups when reaching with their dominant hand. Furthermore, variability of |ΔAE| was greater in the MS-NR group when reaching with their non-dominant hand (*x̄* = 3.1° ± 0.3°) compared to their dominant hand (*x̄* = 1.9° ± 0.3°, *p* < 0.001).

### Individual performance: Offline movement planning and online movement control

In the S2 File we provide an overview of the percentage of participants (S3 and S4 Tables in S2 File), as well as the percentage of trials (S5 Table in S2 File), that fall outside a 95% bandwidth established based on performance of the CTL group. We see that more participants in the MS-R group had difficulty in both the offline planning and online control of their movements compared to participants in the CTL group. Mainly, a greater number of participants in the MS-R group had difficulty consistently initiating their movements (Dominant hand SD RT = 41.7% of participants, (i.e., *n* = 5); Non-dominant hand SD RT = 25.0%, *n* = 3), took longer to complete their movements and were more variable with respect to MT (Dominant hand MT = 33.3%, *n* = 4; Non-dominant hand MT = 25.0%, *n* = 3; Dominant hand SD MT = 50.0%, *n* = 6; Non-dominant hand SD MT = 16.7%, *n* = 2), and demonstrated greater and inconsistent direction errors early on in the trajectory (Dominant hand |IDE| = 58.3%, *n* = 7; Non-dominant hand |IDE| = 33.3%, *n* = 4; Dominant hand |SD IDE| = 41.7%, *n* = 5; Non-dominant hand |SD IDE| = 16.7%, *n* = 2), as well as late in the reach (Dominant hand |EPAE| = 58.3%, *n* = 7; Non-dominant hand |EPAE| = 16.7%, *n* = 2; Dominant hand |SD EPAE| = 50.0%, *n* = 6; Non-dominant hand |SD EPAE| = 16.7%, *n* = 2) compared to participants in the CTL group (8.3% of participants (i.e., *n* = 1)).

We also see that more participants in the MS-NR group had difficulty controlling their movements online compared to participants in the CTL group. In general, a greater number of participants in the MS-NR group reached with a greater peak velocity (Dominant hand PV = 33.3%, *n* = 4; Non-dominant hand PV = 16.7%, *n* = 2), had longer and less consistent trajectories (Dominant hand PL = 25.0%, *n* = 3; Non-dominant hand PL = 41.7%, *n* = 5; Dominant hand SD PL = 16.7%, *n* = 2; Non-dominant hand SD PL = 41.7%, *n* = 5) and demonstrated more inaccurate end positions (Dominant hand SD RE = 33.3%, *n* = 4; Non-dominant hand SD RE = 25.0%, *n* = 3) compared to participants in the CTL group.

## Discussion

In this experiment, we sought to characterize visually guided reaching performance in PwMS who report or do not report experiencing upper limb impairment in order to identify potential differences in offline movement planning and online movement control processes across disability and compared to people without MS. Self-reports of impairment by PwMS further reflected disability levels based on EDSS scores and objective upper limb performance based on the 9-HPT, such that participants in the MS-R group experienced greater neurological disability and performed worse on the 9-HPT compared to participants in the MS-NR group.

We found that all participants were able to perform the visually guided reaching task with their dominant and non-dominant hands, such that the cursor representing their hand position landed on the target and endpoint errors were comparable across groups. Our analysis of offline movement planning and online movement control measures indicated that participants in the MS-R group were less accurate and more inconsistent in their initial reach direction errors compared to participants in the CTL group. As well, participants in the MS-R group were less consistent with respect to the time it took them to initiate and execute their movements compared to participants in the CTL group, as determined by variability in their reaction time and movement time, respectively. Together, these results indicate that PwMS who self-report upper limb impairment experience deficits in both offline planning and online control.

Reaching performance by participants in the MS-NR group also displayed deficits in online control, such that more participants in the MS-NR group were more variable in their performance measures related to online movement control compared to participants in the CTL group (i.e., there was a number of MS-NR participants outside the 95^th^ bandwidth observed in the CTL group). The differences in performance variability between PwMS compared to the CTL group suggests that MS results in more inconsistent movements, requiring PwMS to have to correct for movement errors more often (i.e., engage online control processes) than the CTL group. Thus, PwMS may be more dependent on sensory feedback in order to accurately navigate and interact with objects in their everyday environment.

Other studies that have quantified upper limb performance in PwMS lend support to our proposal that PwMS perform additional online reach corrections compared to control participants without MS [50–52, 59, 61, 62]. In these previous experiments, participants reached to targets in a virtual reality environment such that a participant’s hand was represented by a cursor on the screen and participants were instructed to perform center-out reaching movements to one of eight targets arranged in a circle, as in our task. Results from experiments by Solaro et al. [62] and Pellegrino et al. [59] indicated that PwMS performed less smooth movements and spent a greater proportion of their time navigating to the target compared to control participants. Further analysis by Pellegrino and colleagues revealed that PwMS took longer to complete their movements and had poorer accuracy both early (i.e., 100 ms into the movement) and late in the movement (i.e., at movement end) compared to control participants. Our findings are consistent with these previous results, which suggest that PwMS generally spend additional time navigating to the target, demonstrating impaired online movement control, compared to control participants without MS. Furthermore, the current experiment adds to this literature by identifying deficits in these online control processes in PwMS who report and who do not report upper limb impairment, as well as differ with respect to disability levels as established by the EDSS and objective upper limb performance on the 9-HPT.

### Compensatory strategies

The necessity to make corrective actions in our MS-R group could be attributed to deficits in movement planning from trial-to-trial. Specifically, some of the observed deficits in online movement control in our MS-R group may arise due to impaired offline planning. As previously mentioned, the MS-R group demonstrated greater errors early in the trajectory that would need to be corrected in order for participants to land on the target. These corrections required participants to engage in compensatory strategies (i.e., online movement corrections) in order to complete the task. This notion of MS participants having to engage in compensatory strategies due to poor movement planning is consistent with previous reaching experiments in which PwMS had to complete a Hand to Mouth (HTM) task. In particular, participants were asked to raise their hand to touch their mouth with their fingertips and then return it to the starting position, mimicking eating movements [60, 71–73]. In general, results across these experiments indicated that PwMS experience difficulties in effectively performing the HTM task, as characterized by reduced velocities in the initial movement phase and more time spent in the adjustment phase (i.e., online control) compared to people without MS. The researchers suggest that this combination of impairment in the initial movement and adjustment phase can be considered strategic in PwMS, as reduced velocities enable individuals to use available visual or proprioceptive information to correct their ongoing movements, ensuring accurate localization of the mouth [61, 72]. Collectively, these studies and our current work, suggest that the need to make online corrections by the end of the movement may stem from poorly planned movements.

In contrast to our MS-R group, we did not observe significant deficits in movement planning in our MS-NR group compared to the CTL group. Instead, participants in the MS-NR group tended to achieve higher peak velocities which enabled them to get their hand to the target area more quickly. Despite this reaching strategy, MS-NR participants were faced with difficulties in performing efficient corrections to get their hand on the target. This resulted in longer trajectories and less consistent endpoints with respect to the centre of the target compared to the CTL group. The lack of significant mean performance differences between participants in the MS-NR group compared to the CTL group may be reflective of the variability in performance, and hence reaching strategies engaged within the MS-NR group. Trends for select measures of online movement control generally reflected a number of MS-NR participants falling outside the 95% bandwidth established by performance by age-matched controls. In fact, a greater number of participants in the MS-NR group were more variable in movement duration, had more variable path lengths and more inaccurate and inconsistent end positions compared to individuals in the MS-R and CTL groups. These results suggest that even though participants in the MS-NR group do not report experiencing upper limb impairment, and indeed their performance on the 9-HPT does not differ from CTL, impairment is still present as captured via robotic assessment.

The differences in reaching strategies adopted between PwMS and control participants without MS, may reflect differences in underlying neural activation. Previous literature in people without MS has linked specific premotor and association areas of the cortex to strategic decision making (i.e., offline planning; [74, 75]), while other work has shown that the superior parietal cortex is implicated in movement corrections during execution (i.e., online control; [76–78]). It is assumed that PwMS activate a larger cortical area when carrying out simple and complex upper limb movements compared to control participants, possibly due to widespread tissue damage and inefficient neural activation [79–82]. Thus, it has been suggested that tissue damage in any of the motor areas discussed would require the nervous system to compensate with additional neural activation in order for participants to maintain movement accuracy [61, 83, 84].

### Hand differences

Due to the heterogeneity of MS pathology, impairment often differs in a contralateral manner [20, 85–88]. Thus, our participants completed the visually guided reaching task with both the dominant and non-dominant hands in order to tease out potential differences between limbs. Our findings are in stark contrast with previous experiments [73, 89, 90] that report participants with moderate disability (EDSS < 3.5) demonstrate limited differences in performance between their hands, whereas participants with more severe disability (EDSS > 5.0) exhibit significant differences in performance between their hands. We only observed significant performance differences between the dominant and non-dominant hands in our MS-NR group, who would be classified as experiencing mild to moderate disability based on EDSS scores (EDSS range of 2.5–3.5). Specifically, reaches with the dominant hand were more accurate and were more consistent early on compared to reaches with the non-dominant hand, as demonstrated by lower endpoint angular errors and lower initial reach direction error variability, respectively. These deficits in the non-dominant hand may not be recognized by participants in the MS-R group, as they may be compensating with the dominant hand when completing everyday tasks [85].

### Limitations

In the current experiment, we recruited PwMS who self-report or who do not self-report experiencing upper limb impairment to assess impairment across a range of perceived disability levels. Our MS grouping based on self-reported upper limb impairment corresponded to performance on clinical and objective performance tests, such that we observed significant group differences with respect to EDSS scores (MS-R vs. MS-NR) and performance on the 9-HPT (MS-R vs. MS-NR and CTL). Like the work by Coderre et al. [46] and Simmatis et al. [50], we conducted individual analyses to identify subtle upper limb impairments, before analyzing performance at a group level. The number of participants within each of our groups (i.e., *n* = 12) is similar to previous literature employing a visually-guided reaching task [52, 59, 61]. That said, more recent literature, which has looked to have participants complete a battery of robotic tasks, has recruited additional participants (e.g., *n*_PwMS_ = 43; [50]), and thus we refrain from making claims with respect to implications for the design of rehabilitation protocols across disability status. Future research should further consider the inclusion of neuroimaging to explore the complex relationship between neurological damage and compensatory strategies in MS, as identified by measures of offline planning and online control.

## Conclusion

To our knowledge, this is the first experiment to characterize and explore upper limb visually guided reaching performance in PwMS who report or do not report experiencing upper limb impairment. We found that PwMS who report upper limb impairment are less consistent in initiating and executing their movements, as well as possess greater and more variable initial directional errors than people without MS, when reaching with both their dominant and non-dominant hands. Nevertheless, such errors and variability did not prevent participants from completing the visually guided reaching task, and accurately landing their hand on the target. This is likely because PwMS who report upper limb impairment compensated for deficits in offline movement planning and possess an intact ability to make online corrective movements throughout the reach. Overall, performance by PwMS who do not report upper limb impairment did not significantly differ from both PwMS who report upper limb impairment as well as people without MS, at a group level of analyses. However, several PwMS who do not report upper limb impairment showed deficits in online movement control, highlighting the variability in performance within this group. Thus, through the exploration of visually guided reaches performed by PwMS, we were able to detect clear deficits in movement control processes in PwMS compared to control participants regardless of self-reports of upper limb impairment. Moving forward, an understanding of how PwMS, who report and do not report upper limb impairment, perform visually guided reaches can be used to prevent the adoption of maladaptive reaching strategies [50, 91].

## Supporting information

S1 File Performance measures. Mean and standard error of the mean for offline planning (S1 Table in S1 File) and online control (S2 Table in S1 File) measures.
(DOCX)

S2 File Performance compared to control participants. Percentage of participants (S3 and S4 Tables in S2 File) and trials (S5 Table in S2 File) beyond the 95^th^ percentile in the CTL group for offline planning and online control measures.
(DOCX)
