# Supplementary material for: Assessing visually guided reaching in people with multiple sclerosis with and without self-reported upper limb impairment
Source: PLoS One. 2022 Jan 21;17(1):e0262480. doi: 10.1371/journal.pone.0262480 (PMC8782348; doi:10.1371/journal.pone.0262480)
Supplement: S1 File — Mean and standard error of the mean for offline planning (S1 Table in S1 File) and online control (S2 Table in S1 File) measures. (DOCX) [file pone.0262480.s001.docx]

# Supplementary File 1

## Performance measures

**S1 Table. Mean and standard error of the mean (in brackets) for offline planning measures.** Reaction time (RT), reaction time variability (SD RT), peak velocity (PV), peak velocity variability (SD PV), proportional time to peak velocity (pTTPV), proportional time to peak velocity variability (SD pTTPV), absolute initial reach direction error (|IDE|), and absolute initial reach direction error variability (|SD IDE|) are presented for all groups.

| Offline Planning Measures | | | | | | | | | | | | | | | | | |
| --- | --- | --- | --- | --- | --- | --- | --- | --- | --- | --- | --- | --- | --- | --- | --- | --- | --- |
|  | | RT  (ms) | | SD RT  (ms) | | PV  (m/s) | | SD PV (m/s) | | pTTPV (%) | | | SD pTTPV  (%) | \|IDE\|  (°) | | | \|SD IDE\|  (°) |
| Non-dominant Hand | | |  | | | |  | | | |  | | | |  | | |
| CTL | | 335.6  (24.4) | | 56.0*  (7.1) | | 0.23  (0.03) | | 0.04  (0.01) | | 50.9  (2.6) | | | 14.1  (1.0) | 4.8*  (0.6) | | | 4.3  (0.7) |
| MS-NR | | 342.8  (24.4) | | 66.4  (7.1) | | 0.25  (0.25) | | 0.06  (0.01) | | 53.2  (2.6) | | | 15.6  (1.0) | 6.2  (0.6) | | | 6.1^†^  (0.7) |
| MS-R | | 405.6  (24.4) | | 79.3*  (7.1) | | 0.24  (0.25) | | 0.04  (0.01) | | 52.7  (2.6) | | | 15.4  (1.0) | 6.5*  (0.6) | | | 5.6  (0.7) |
| Dominant Hand |  | | | |  | | | |  | | |  | | | |  | |
| CTL | | 322.6  (20.8) | | 53.2*  (8.5) | | 0.24  (0.20) | | 0.04  (0.01) | | 52.5  (2.4) | | | 13.8  (0.9) | 4.2*  (0.5) | | | 3.6*  (0.5) |
| MS-NR | | 339.5  (20.8) | | 56.0  (8.5) | | 0.25  (0.20) | | 0.04  (0.01) | | 54.6  (2.4) | | | 13.8  (0.9) | 5.0  (0.5) | | | 4.1^†^  (0.5) |
| MS-R | | 399.3  (20.8) | | 85.2*  (8.5) | | 0.22  (0.20) | | 0.05  (0.01) | | 54.4  (2.4) | | | 143.  (0.9) | 6.4*  (0.5) | | | 5.7*  (0.5) |

*Asterisks* (*) represent significant differences between MS-R and CTL groups.

*Dagger* (†) represent a significant difference between Hands (*p* < 0.05).

**S2 Table. Mean and standard error of the mean (in brackets) for online control measures.** Movement time (MT), movement time variability (SD MT), resultant error (RE), resultant error variability (SD RE), absolute endpoint angular error (|EPAE|), absolute endpoint angular error variability (|SD EPAE|), absolute change in angular error (|ΔAE|), absolute change in angular error variability (|SD ΔAE|), path length (PL) and path length variability (SD PL) are presented for all trials.

| Online Control Measures | | | | | | | | | | | | | | |
| --- | --- | --- | --- | --- | --- | --- | --- | --- | --- | --- | --- | --- | --- | --- |
|  | MT  (ms) | SD MT  (ms) | RE  (cm) | | SD RE (cm) | | \|EPAE\|  (°) | | \|SD EPAE\|  (°) | | \|ΔAE\|  (°) | \|SD ΔAE\|  (°) | PL  (cm) | SD PL (cm) |
| Non-dominant Hand | | | |  | |  | |  | |  | |  |  |  |
| CTL | 673.8  (48.9) | 130.5*  (13.7) | 1.4  (0.1) | | 0.4  (0.1) | | 2.6^†^  (0.4) | | 2.2  (0.5) | | 3.5^†^  (0.4) | 3.7  (0.5) | 9.4  (0.3) | 0.9  (0.4) |
| MS-NR | 724.4  (48.9) | 154.9  (13.7) | 1.5  (0.1) | | 0.5  (0.1) | | 3.5^†^  (0.4) | | 3.1  (0.5) | | 4.3^†^  (0.4) | 5.2^†^  (0.5) | 10.1  (0.3) | 2.0  (0.4) |
| MS-R | 813.4  (48.9) | 169.2*  (13.7) | 1.3  (0.1) | | 0.4  (0.1) | | 3.4^†^  (0.4) | | 3.2  (0.5) | | 4.8^†^  (0.4) | 4.9  (0.5) | 9.8  (0.3) | 1.3  (0.4) |
| Dominant Hand | | | |  | |  | |  | |  | |  |  |  |
| CTL | 655.6  (46.0) | 116.5*  (14.4) | 1.4  (0.1) | | 0.3  (0.1) | | 2.0^†^  (0.2) | | 1.7  (0.3) | | 3.3^†^  (0.5) | 3.1  (0.4) | 9.4  (0.2) | 1.0  (0.3) |
| MS-NR | 698.8  (46.0) | 126.2  (14.4) | 1.5  (0.1) | | 0.4  (0.1) | | 2.8^†^  (0.2) | | 2.2  (0.3) | | 3.2^†^  (0.5) | 3.2^†^  (0.4) | 9.8  (0.2) | 1.4  (0.3) |
| MS-R | 794.4  (46.0) | 177.6*  (14.4) | 1.4  (0.1) | | 0.4  (0.1) | | 2.8^†^  (0.2) | | 2.5  (0.3) | | 4.7^†^  (0.5) | 4.6^§^  (0.4) | 9.7  (0.2) | 1.1  (0.3) |

*Asterisks* (*) represent significant differences between MS-R and CTL groups.

*Section* (§) represent a significant difference between the MS-R group and the two other groups: MS-NR and CTL.

*Dagger* (†) represent a significant difference between Hands (*p* < 0.05).
