# Supplementary material for: Assessing visually guided reaching in people with multiple sclerosis with and without self-reported upper limb impairment
Source: PLoS One. 2022 Jan 21;17(1):e0262480. doi: 10.1371/journal.pone.0262480 (PMC8782348; doi:10.1371/journal.pone.0262480)
Supplement: S2 File — Percentage of participants (S3 and S4 Tables in S2 File) and trials (S5 Table in S2 File) beyond the 95th percentile in the CTL group for offline planning and online control measures. (DOCX) [file pone.0262480.s002.docx]

# Supplementary File

## Performance compared to control participants

In Tables S3-S5, we compare individual MS participant performance to the 95^th^ percentile bandwidth observed in the CTL group for each of our measures of offline planning and online control (mean and variability). Thus, we sought to determine the percentage of MS participants who performed unlike 95% of the participants within the CTL group. Similar to work by Coderre et al., (2010) in patients post stroke, as well as Simmatis et al., (2020) in PwMS, we categorized performance falling above the 95^th^ percentile observed in the CTL group as an indication of upper limb impairment.

We found that performance by a large number of participants in the MS-R group fell outside the 95^th^ percentile. Specifically, more participants in the MS-R group took longer to initiate (i.e., RT) and execute (i.e., MT) a movement, spent a longer proportion of the movement early in the trajectory (i.e., pTTPV) and were generally less accurate throughout the reach (i.e., |IDE| and |EPAE|) compared to participants in the CTL and MS-NR groups. On the other hand, more participants in the MS-NR group were faster (i.e., PV), performed longer, inconsistent trajectories (i.e., PL and SD PL) and were less consistent in their end position (i.e., SD RE) compared to the participants in the CTL and MS-R groups.

A similar assessment was performed with respect to individual trials, such that we sought to determine the percentage of trials across all MS participants that were unlike 95% of the trials performed by the CTL group (Table S5). We identified similar trends across measures as discussed in Tables S3 and S4, such that measures of planning and control differed between PwMS and participants in the CTL group. MS-R participants had a greater number of trials that were outside the 95^th^ percentile of the CTL group for measures of planning (i.e., RT, |IDE| and pTTPV), whereas MR-NR participants demonstrated poorer performance across measures of control (i.e., |EPAE|, RE, and PL). These results present an opportunity to establish individual participants who performed poorly (i.e., above the 95^th^ percentile). As well, the results reveal differences in performance related to movement execution in the MS-NR group compared to the CTL group, whereas we see greater deficits in movement planning in MS-R compared to control participants.

**S3 Table. Percentage of participants that fall outside the 95^th^ percentile bandwidth observed in the CTL group for performance measures related to offline movement planning.** Reaction time (RT), reaction time variability (SD RT), peak velocity (PV), peak velocity variability (SD PV), proportional time to peak velocity (pTTPV), proportional time to peak velocity variability (SD pTTPV), absolute initial reach direction errors (|IDE|), and absolute initial reach direction error variability (|SD IDE|) are presented for all trials.

|  | RT | SD RT | PV | SD PV | pTTPV | SD pTTPV | \|IDE\| | \|SD IDE\| |
| --- | --- | --- | --- | --- | --- | --- | --- | --- |
| Non-Dominant Hand | | | | | | | | |
| CTL | 8.3 % | 8.3 % | 8.3 % | 8.3 % | 8.3 % | 0.0 % | 8.3 % | 8.3 % |
| MS-NR | 0.0 % | 8.3 % | 16.7 % | 16.7 % | 16.7 % | 16.7 % | 25.0 % | 33.3 % |
| MS-R | 8.3 % | 25.0 % | 8.3 % | 8.3 % | 41.7 % | 25.0 % | 33.3 % | 16.7 % |
| Dominant Hand | | | | | | | | |
| CTL | 8.3 % | 8.3 % | 8.3 % | 8.3 % | 8.3 % | 8.3 % | 8.3 % | 8.3 % |
| MS-NR | 8.3 % | 8.3 % | 33.3 % | 16.7 % | 16.7 % | 0.0 % | 33.3 % | 18.7 % |
| MS-R | 41.7 % | 41.7 % | 8.3 % | 25.0 % | 16.7 % | 8.3 % | 58.3 % | 41.7 % |

**S4 Table. Percentage of participants that fall outside the 95^th^ percentile bandwidth observed in the CTL group for performance measures related to online movement control.** Movement time (MT), movement time variability (SD MT), resultant error (RE), resultant error variability (SD RE), absolute endpoint angular error (|EPAE|), absolute endpoint angular error variability (|SD EPAE|), absolute change in angular error (|ΔAE|), absolute change in angular error variability (|SD ΔAE|), path length (PL), and path length variability (SD PL) are presented for all trials.

|  | MT | SD MT | | RE | | SD RE | | \|EPAE\| | | \|SD EPAE\| | | \|ΔAE\| | | \|SD ΔAE\| | | PL | | SD PL |  |
| --- | --- | --- | --- | --- | --- | --- | --- | --- | --- | --- | --- | --- | --- | --- | --- | --- | --- | --- | --- |
| Non-Dominant Hand | | |  | |  | |  | |  | |  | |  | |  | |  | |  |
| CTL | 8.3 % | 8.3 % | | 8.3 % | | 8.3 % | | 8.3 % | | 8.3 % | | 8.3 % | | 8.3 % | | 8.3 % | | 8.3 % |  |
| MS-NR | 16.7 % | 25.0 % | | 8.3 % | | 25.0 % | | 25.0 % | | 8.3 % | | 16.7 % | | 25.0 % | | 41.7 % | | 41.7 % |  |
| MS-R | 25.0 % | 16.7 % | | 8.3 % | | 16.7 % | | 16.7 % | | 16.7 % | | 25.0 % | | 33.3 % | | 8.3 % | | 16.7 % |  |
| Dominant Hand | | |  | |  | |  | |  | |  | |  | |  | |  | |  |
| CTL | 8.3 % | 8.3 % | | 8.3 % | | 8.3 % | | 8.3 % | | 8.3 % | | 8.3 % | | 8.3 % | | 8.3 % | | 8.3 % |  |
| MS-NR | 16.7 % | 25.0 % | | 16.7 % | | 33.3 % | | 50.0 % | | 25.0 % | | 0.0 % | | 8.3 % | | 25.0 % | | 16.7 % |  |
| MS-R | 33.3 % | 50.0 % | | 16.7 % | | 25.0 % | | 58.3 % | | 50.0 % | | 8.3 % | | 50.0 % | | 25.0 % | | 16.7 % |  |

**S5 Table. Percentage of trials that fall outside the 95^th^ percentile bandwidth observed in the CTL group.** Reaction time (RT), peak velocity (PV), time to peak velocity (TTPV), absolute initial reach direction errors (|IDE|), movement time (MT), resultant error (RE), absolute endpoint angular error (|EPAE|), absolute change in angular error (|ΔAE|), and path length (PL) are presented for all trials.

|  | Offline Planning Measures | | | | | | |  | Online Control Measures | | | | |
| --- | --- | --- | --- | --- | --- | --- | --- | --- | --- | --- | --- | --- | --- |
|  | RT | PV | | TTPV | | \|IDE\| | |  | MT | RE | \|EPAE\| | \|ΔAE\| | PL |
| Non-dominant Hand | | |  | |  | |  |  |  |  |  |  |  |
| CTL | 4.9 % | 4.9 % | | 4.9 % | | 5.1 % | |  | 4.9 % | 4.9 % | 5.1 % | 4.9 % | 4.9 % |
| MS-NR | 4.0 % | 10.7 % | | 8.5 % | | 8.9 % | |  | 10.7 % | 9.5 % | 11.1 % | 8.1 % | 12.6 % |
| MS-R | 11.1 % | 5.6 % | | 19.4 % | | 9.5 % | |  | 17.8 % | 3.4 % | 9.0 % | 10.9 % | 9.9 % |
| Dominant Hand | | |  | |  | |  |  |  |  |  |  |  |
| CTL | 5.1 % | 5.1 % | | 5.1 % | | 5.1 % | |  | 5.1 % | 5.1 % | 5.1 % | 5.1 % | 5.1 % |
| MS-NR | 9.1 % | 8.2 % | | 7.4 % | | 8.7 % | |  | 9.9 % | 10.2 % | 13.0 % | 4.9 % | 13.7 % |
| MS-R | 24.1 % | 4.9 % | | 16.8 % | | 16.4 % | |  | 19.3 % | 7.2 % | 12.8 % | 14.5 % | 15.4 % |
